# Supplementary material for: DMRforPairs: identifying Differentially Methylated Regions between unique samples using array based methylation profiles
Source: BMC Bioinformatics. 2014 May 15;15:141. doi: 10.1186/1471-2105-15-141 (PMC4046028; doi:10.1186/1471-2105-15-141)
Supplement: Additional file 2 — DMRforPairs output for the comparison of A431-MCF7 and NA17018-NA17105. Please start from the HTML files in each folder. Available via the BMC Bioinformatics website. [file 1471-2105-15-141-S2.zip › 1394847754114233_MOESM2_ESM/A431_MCF7/figures/12590.pdf]

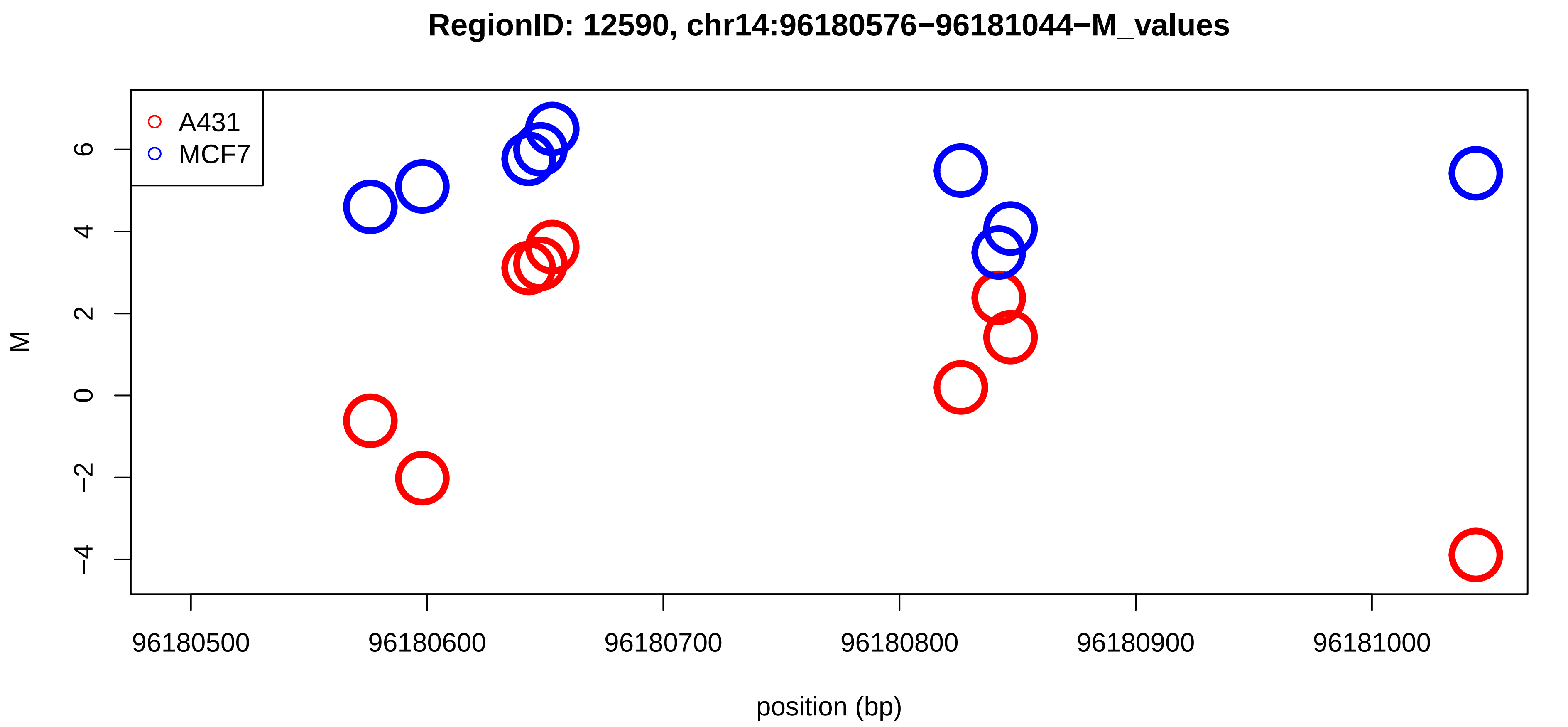
RegionID: 12590, chr14:96180576−96181044−M_values
A431
MCF7
M
position (bp)
96180500
96180600
96180700
96180800
96180900
96181000

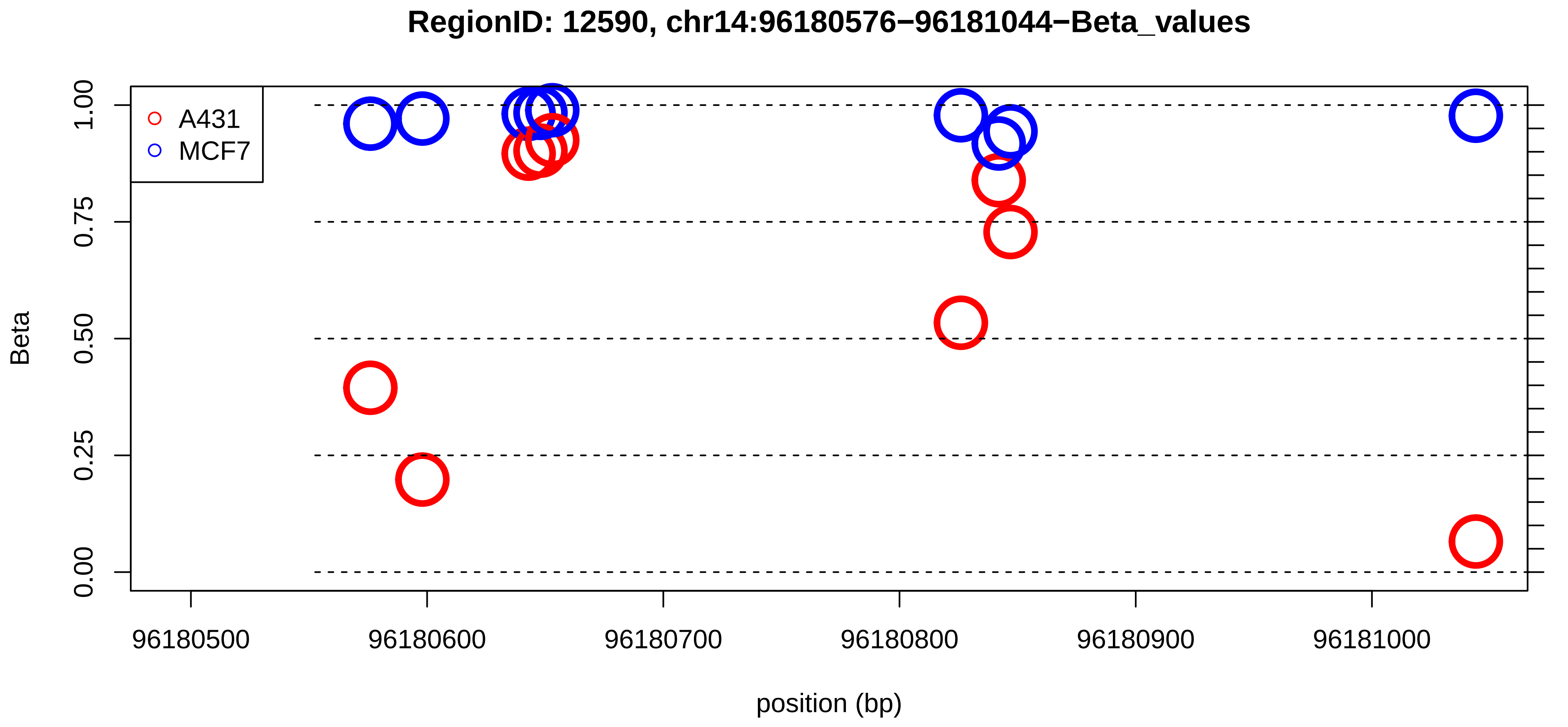
RegionID: 12590, chr14:96180576−96181044−Beta_values
A431
MCF7
Beta
position (bp)
96180500
96180600
96180700
96180800
96180900
96181000

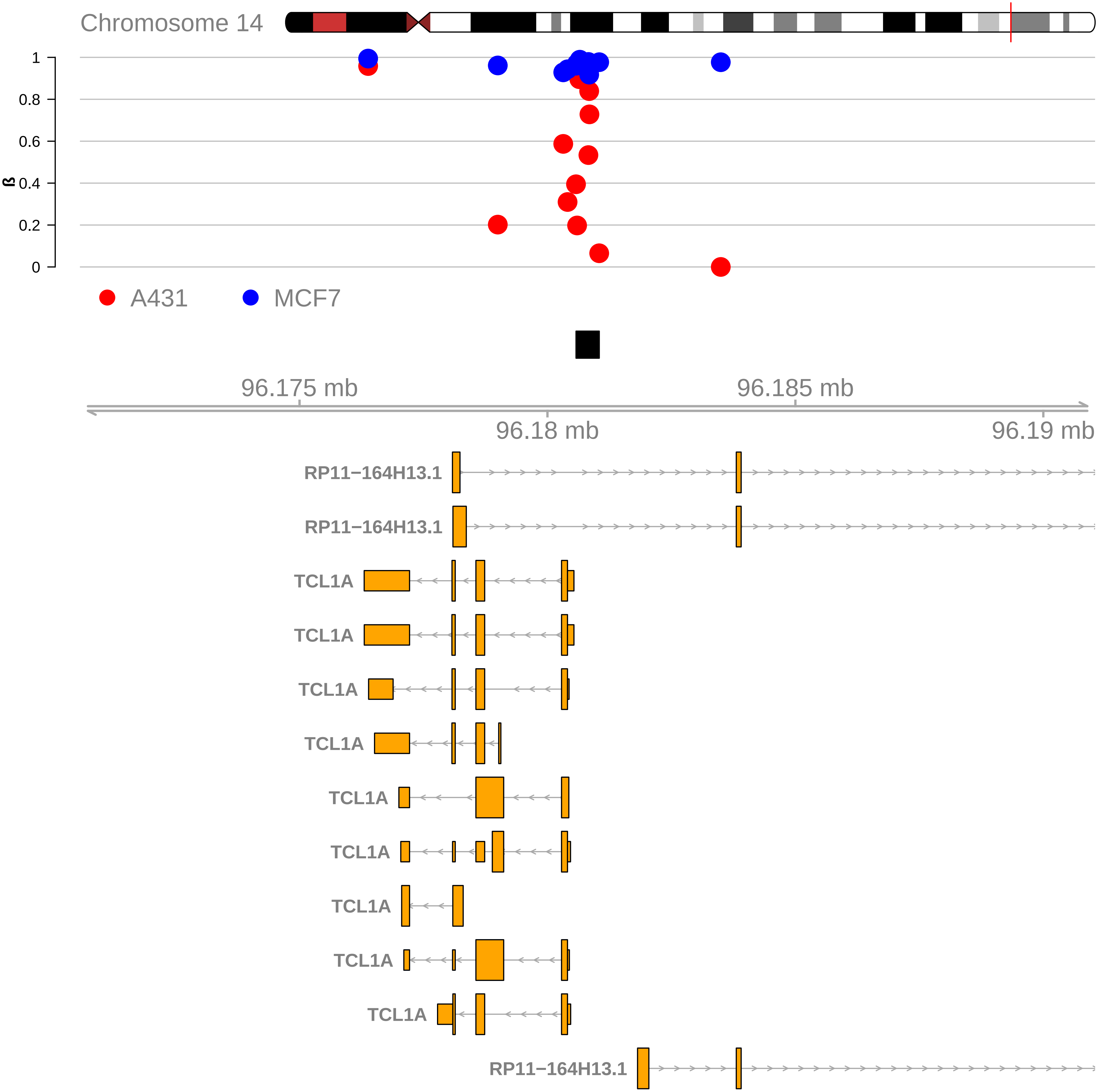
Chromosome 14
1
0.8
0.6
ß 0.4
0.2
0
A431
MCF7
96.175 mb
96.185 mb
96.18 mb
96.19 mb
RP11−164H13.1
RP11−164H13.1
TCL1A
TCL1A
TCL1A
TCL1A
TCL1A
TCL1A
TCL1A
TCL1A
TCL1A
RP11−164H13.1
